# Characterization of Niemann-Pick Type C2 Protein Expression in Multiple Cancers Using a Novel NPC2 Monoclonal Antibody

**DOI:** 10.1371/journal.pone.0077586

**Published:** 2013-10-17

**Authors:** Yi-Jen Liao, Meng-Wei Lin, Chia-Hung Yen, Yu-Ting Lin, Chung-Kwe Wang, Shiu-Feng Huang, Kuan-Hsuan Chen, Ching-Ping Yang, Tzu-Lang Chen, Ming-Feng Hou, Yi-Ming Arthur Chen

**Affiliations:** 1 School of Medical Laboratory Science and Biotechnology, College of Medical Science and Technology, Taipei Medical University, Taipei, Taiwan; 2 Institute of Clinical Medicine, National Yang-Ming University, Taipei, Taiwan; 3 Graduate Institute of National Products, College of Pharmacy, Kaohsiung Medical University, Kaohsiung, Taiwan; 4 Department of Microbiology, College of Medicine, Kaohsiung Medical University, Kaohsiung, Taiwan; 5 Department of International Medicine, Taipei City Hospital Ranai Branch, Taipei, Taiwan; 6 Division of Molecular and Genomic Medicine, National Health Research Institute, Miaoli, Taiwan; 7 Kaohsiung Municipal Ta-Tung Hospital, Kaohsiung, Taiwan; Yokohama City University School of Medicine, Japan

## Abstract

Niemann-Pick Type C2 (NPC2) plays an important role in the regulation of intracellular cholesterol homeostasis via direct binding with free cholesterol. However, little is known about the significance of NPC2 in cancer. In this study, we have pinpointed the impact of various different cancers on NPC2 expression. A series of anti-NPC2 monoclonal antibodies (mAbs) with the IgG2a isotype were generated and peptide screening demonstrated that the reactive epitope were amino acid residues 31-40 of the human NPC2 protein. The specificity of these mAbs was confirmed by Western blotting using shRNA mediated knock-down of NPC2 in human SK-Hep1 cells. By immunohistochemical staining, NPC2 is expressed in normal kidney, liver, breast, colon, lung, esophageal, uterine cervical, pancreatic and stomach tissue. Strong expression of NPC2 was found in the distal and proximal convoluted tubule of kidney and the hepatocytes of liver. Normal esophageal, uterine cervical, pancreatic, stomach, breast, colon and lung tissue stained moderately to weakly. When compared to their normal tissue equivalents, NPC2 overexpression was observed in cancers of the breast, colon and lung. Regarding to breast cancer, NPC2 up-regulation is associated with estrogen receptor (-), progesterone receptor (-) and human epidermal growth factor receptor (+). On the other hand, NPC2 was found to be down-regulated in renal cell carcinoma, liver cirrhosis and hepatoma tissues. By antigen-capture enzyme immunoassay ELISA, the serum NPC2 is increased in patients with cirrhosis and liver cancer. According to western blot data, the change of glycosylated pattern of NPC2 in serum is associated with cirrhosis and liver cancer. To the best of our knowledge, this is the first comprehensive immunohistochemical and serological study investigating the expression of NPC2 in a variety of different human cancers. These novel monoclonal antibodies should help with elucidating the roles of NPC2 in tumor development, especially in liver and breast cancers.

## Introduction

Niemann-Pick Type C2 (NPC2) protein is a small soluble glycoprotein that contains a nineteen amino acids signal peptide. The protein was first characterized as a major secretory protein in the human epididymis [1]. NPC2 plays an important role in the regulation of intracellular cholesterol homeostasis via direct binding with free cholesterol [2]. A deficiency in NPC2 results in the accumulation of free cholesterol in the lysosome [3]. Analysis of NPC2 mRNA by Northern blotting has revealed a single transcript of 0.9 kb in all tissues examined, with the highest mRNA levels in the testis, kidney and liver [4]. The mature human NPC2 protein consists of 132 amino acids and is expressed as different isoforms; these vary in size from 19 to 23 kD in a tissue-specific fashion [5,6]. Recently, we showed that NPC2 acts coordinately with glycine N-methyltransferase to regulate hepatic cholesterol homeostasis and fatty liver disease progression [7]. Furthermore, NPC2 is essential for papillae formation and modulates papillary growth [8]. NPC2 is also expressed in alveolar epithelial type II cells from the lung [9]. Since NPC2 negatively regulates ERK1/2 mitogen activated protein kinase (MAPK) phosphorylation in fibroblast cells [10], a disturbance in NPC2 expression may be associated with important human diseases including cancer. However, the expressions of NPC2 in human cancers have not been explored in detail. Therefore, our research goals were (a) to develop a panel of monoclonal antibodies (mAbs) targeted against the NPC2 protein and (b) to characterize their properties and possible clinical applications. By the use of immunohistochemical staining, strong levels of expression of NPC2 were found in the distal and proximal convoluted tubule of kidney and in the hepatocytes of liver. The expression of NPC2 was found to be up-regulated in human breast, colon and lung cancers, while, in contrast, there was down-regulation of NPC2 expression in kidney and liver cancers. Finally, we further demonstrated that the up-regulation of NPC2 is correlated with the status of estrogen receptor (ER), progesterone receptor (PR) and human epidermal growth factor receptor (HER-2) expression. In addition, dysregulation of sera NPC2 is associated with liver cirrhosis and hepatocellular carcinoma (HCC).

## Materials and Methods

### Generation of monoclonal antibodies against NPC2

**Figure 1:**
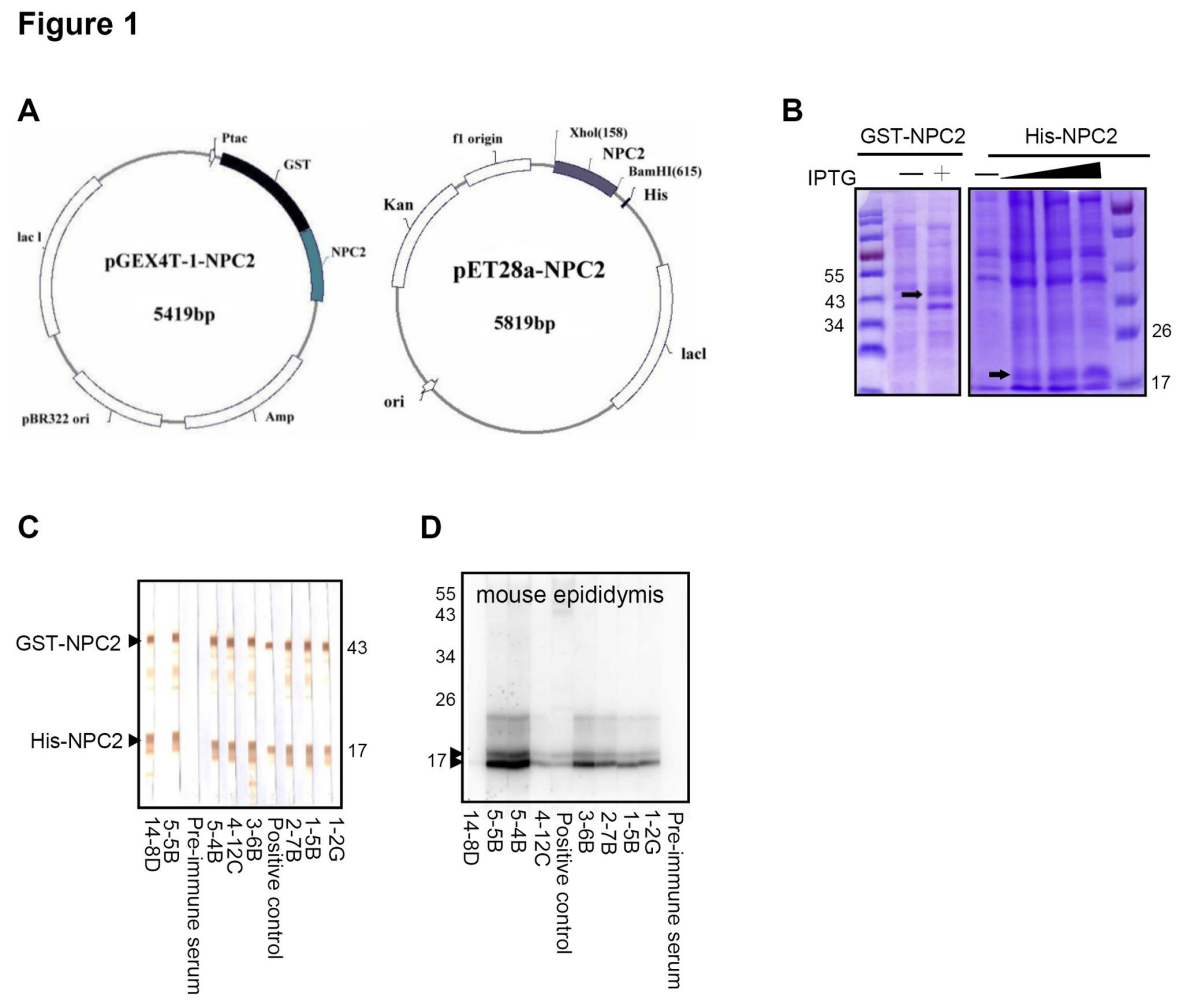
Development of NPC2 monoclonal antibodies. (A) Construction of the expression plasmids of GST-NPC2 and His-NPC2. (B) Bacterial NPC2 fusion proteins were expressed in BL21 cells and then induced using 1 mM IPTG. SDS-PAGE and Coomassie blue staining were used to show the molecular weights of the two proteins were approximately 43.3 kD for GST-NPC2 and 17 kD for His-NPC2. (C) Western blot analysis using the mAbs (14-8D, 5-5B, 5-4B, 4-12C, 3-6B, 2-7D, 1-5B and 1-2G) against GST-NPC2 and His-NPC2. (D) Western blot analysis using the mAbs against mouse epididymis.

To generate a series of mAbs against NPC2, purified GST-NPC2 or purified His-NPC2 (Figure 1A) recombinant protein (RP) were mixed with Freund’s complete adjuvant (for the initial immunization) or incomplete Freund’s adjuvant (for the booster injections) (Sigma Co., St. Louis, Mo., USA) and the resultant mixture was used as an immunogen. His-NPC2 RP was used as a screening antigen for antibody arose by GST-NPC2 RP. Mouse mAbs were produced by the hybridoma technique [11]. The hybridomas were dispensed into six 96-well plates and cultured in selected medium [12]. The culture supernatants were screened using an enzyme immunoassay with GST-NPC2 RP and His-NPC2 RP as the antigens. Hybridoma cells that have a high optic density by enzyme immunoassay were confirmed by Western blot assay immediately. Each well of cells that produced a positive result was sub-cloned into a 96-well plate with a cell density of 0.5 cell per well. Resulting single clones with a positive result were then inoculated at a dosage of 2 x 10^6^ into a BALB/c mouse that had been primed with 0.5 ml of incomplete adjuvent (Sigma) previously. Monoclonal antibody was purified from the mouse ascetic fluid using protein G plus protein A Agrose (Calbiochem, San Diego, CA, USA) and then concentrated by Vivaspin 20 (GE Healthcare). The isotype of each mAb was determined using Mouse Monoclonal Antibody Isotyping Reagents (Sigma).

### Cells and plasmids

SK-Hep1 cells, purchased from ATCC, were cultured in DMEM (Gibco BRL, Grand Island, NY) with 10% heat-inactivated fetal bovine serum (HyClone, Logan, UT, USA), penicillin (100 U/ml), streptomycin (100 μg/ml), nonessential amino acids (0.1 mM), and L-glutamine (2 mM) in a humidified incubator with 5% CO_2_. Plasmid DNA was transfected using TurboFect™ Reagent (Fermentas, Hanover, MD, USA). The primer sequences and restriction enzymes used for the full-length and different lengths of human NPC2 genes have been previously descried by Liao, et al [7]. Plasmid encoding shRNA for NPC2 (TRCN0000029323) was obtained from the National RNAi Core Facility (Academia Sinica, Taiwan).

### Human samples

The HCC tissue slides (n=50) and sera (n=165) were obtained from the Taiwan Liver Cancer Network (TLCN) and Taipei City Hospital. The clinical features of these patients are shown in Table S1 and Table S2. Healthy control and patients with chronic hepatitis infection, fatty liver, cirrhosis and HCC had been recruited between 2008 and 2010 from the Department of International Medicine, Taipei City Hospital, Ran-Ai Branch, Taipei, Taiwan and TLCN. Total 33 invasive breast cancer subjects were identified in the Kaohsiung Municipal Ta-Tung Hospital. We processed all tissue samples under the same conditions. The Ethics Committee of the Institutional Review Board of National Yang-Ming University (IRB No. 1000041) and Taipei City Hospital Institutional Review Board (IRB No. TCHIRB980509) approved the clinical investigations, and all subjects gave written informed consent. Diagnosis of HCC was confirmed by histological examination. The breast (BR721), colon (CO803), lung (LC1006), kidney (KD991), multiple cancer (BCN721), multiple diseases of liver (LV1201) and liver cirrhosis and hepatitis (LV805) tissues arrays were purchased from Biomax, US. Clinical and pathological information on the individual cancer samples are available and were obtained from the array manufacturer.

### Peptide screening, Western blotting and immunohistochemical (IHC) staining

An ELISA plate coated with peptides was used to map epitopes of the various anti-NPC2 mAbs. These peptides (each with a length of 10 a.a.) covered the complete length of the 1-40 a.a. region of the NPC2 protein. For Western blotting, cellular or mouse epididymis proteins were separated by SDS-PAGE. phospho-ERK1/2 and ERK1/2; phospho-p38 and p38; phospho-JNK and JNK were purchased from Cell Signaling Technology (Beverly, MA, USA). Immunoblotting signals were normalized using α-tubulin or β-actin and quantified by densitometric scanning. IHC detection of the NPC2 protein was performed using NPC2 monoclonal Ab (3-6B) at a dilution of 1:200 [7]. Paraffin-embedded tissue sections were incubated with the primary antibody and detected using Universal LSAB^TM^2 kit (DakoCytomation) according to the manufacturer’s instructions.

### Antigen-capture enzyme immunoassay ELISA

Briefly, 100 µl of polyclonal NPC2 antibody (5 µg/ml) in 0.1 M carbonate buffer (pH 9.6) were added to each well of a 96-well microtiter plate (Corning Costar, Acton, MA) and incubated at 4°C overnight. Then, blocking with 5% BSA in PBST (PBS buffer containing 0.05% Tween) at 37°C for 2hrs. Each patient’s serum (100 µl at a 1:1 dilution) was added to 96 well-plates and incubated at 37°C for 1hr. After three extensive washings with PBST, monoclonal NPC2 antibody (1:1000 dilution) was added and incubated at 37°C for 1hr, followed by incubation with 200 µl substrate (0.015% o-phenylenediamine dihydrochloride) (Sigma-Aldrich) for another 30 min at 37°C. Reactions were stopped by the addition of 3N HCl; absorbance was measured with a spectrophotometer at 490 nm.

### Glycosidase Digestion

Sixty micrograms of liver proteins from human sera were incubated with or without N-glycosidase F (PNGase F) according to the manufacturer’s instruction (NE Biolabs, Beverly, MA). The reaction products were subjected to western blot analysis.

### Statistical analyses

Chi-square goodness of fit, nonparametric Wilcoxon Signed Ranks test or Mann-Whitney U test were used to evaluate the association between NPC2 expression and clinical diseases. A *p* value of ≦0.05 was considered to be statistically significant.

## Results

### Generation and characterization of mAbs against NPC2

In order to develop NPC2 mAbs that will detect NPC2 expression in different cell lines and tissues, we initially cloned a full NPC2 fragment and expressed the GST-NPC2 and His-NPC2 fusion proteins in a bacterial system (Figure 1A). As shown in Figure 1B, the GST-NPC2 and His-NPC2 recombinant proteins, with sizes of 43.3 and 17 kD, respectively, appeared in the IPTG-induced bacterial lysates. The purified GST-NPC2 and His-NPC2 recombinant proteins were used as the antigens during the generation of mAbs against NPC2. In total, eight mAbs (14-8D, 5-5B, 5-4B, 4-12C, 3-6B, 2-7B, 1-5B and 1-2G) were eventually generated and found to react with the GST-NPC2 and His-NPC2 recombinant proteins (Figure 1C). Since NPC2 is a major secretory protein expressed in epididymal fluid [1], we applied our NPC2 mAbs to detect NPC2 expression in mouse epididymis using Western blotting. As shown in Figure 1D, NPC2 was detected in total epididymis extracts as protein bands of approximately 16 to 22 kD. Isotype determination identified all mAbs as belonging to the IgG2a isotype and their light chains were identified as κ chains.

**Figure 2:**
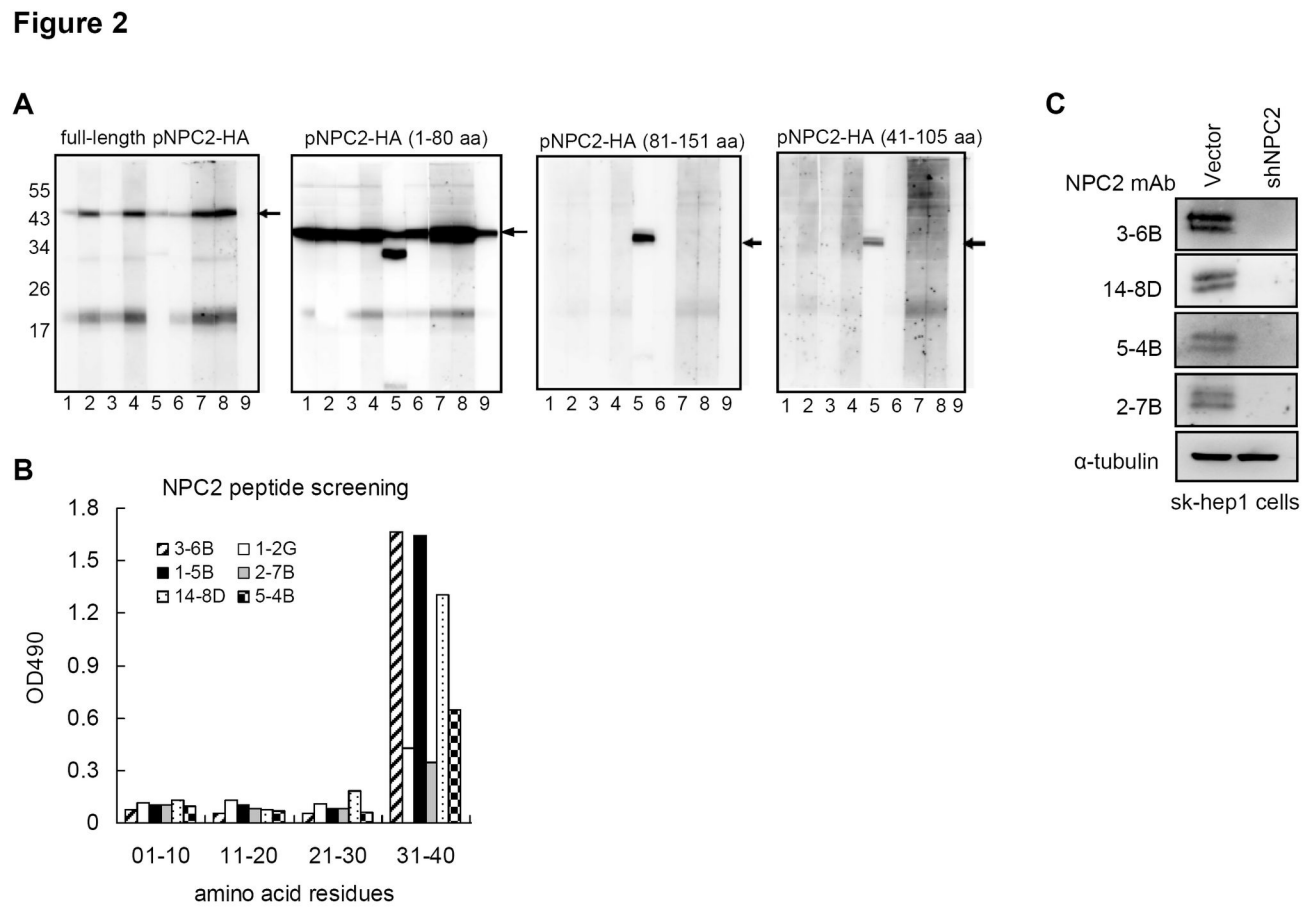
Characterization of NPC2 monoclonal antibodies. (A) Epitope region mapping of the monoclonal NPC2 antibodies. Different length of pNPC2-HA including full-length pNPC2-HA, N-terminal half (1-80 a.a.), C-terminal half (81-151 a.a.) and 41-105 a.a. were transfected into 293T cells and 24 hrs later were harvested for Western blot analysis. Lane 1, 1-2G; Land 2, 1-5B; Lane 3, 2-7B; Lane 4, 3-6B; Lane 5, mAb-HA; Lane 6, 4-12C; Lane 7, 5-4B, Lane 8, 5-5B; Lane 9, 14-8D. (B) An ELISA plate coated with four peptides covering the 1-40 a.a. region of NPC2 protein was used for epitope mapping. Representative results from the mAbs (14-8D, 5-4B, 3-6B, 2-7D, 1-5B and 1-2G) are shown. (C) The knockdown effect of shNPC2 on SK-Hep1 cells was detected using anti-NPC2 mAbs (3-6B, 14-8D, 5-4B and 2-7B).

To map the reactive region recognized by the NPC2 mAbs, we transfected a separate set of plasmids expressing different lengths of NPC2-HA (including full-length NPC2 [1-151 a.a.], domain A and B [1-80 a.a.], domain B and C [40-105 a.a.], and domain C and D [81-151 a.a.]) for 24 hrs. As shown in Figure 2A, all eight NPC2 mAbs can recognize full length and the N-terminal half (1-80 a.a.) of pNPC2-HA. While, fragments containing the 41-105 a.a. region of NPC2 and the C-terminal half (81-151 a.a.) of NPC2 did not react with the NPC2 mAbs. These findings suggest that all of the NPC2 mAbs recognize amino acids 1-40 of NPC2 protein. To further map the reactive epitopes recognized by the NPC2 mAbs, an ELISA was performed using a peptide panel consisting of four peptides covering the amino acids 1-40 of NPC2 protein. Our results indicate that only one peptide (amino acids 31-40) was recognized by six of the selected NPC2 mAbs (14-8D, 5-4B, 3-6B, 2-7B, 1-5B and 1-2G) (Figure 2B). As shown in Figure 2C, the sensitivity and specificity of the NPC2 mAbs (3-6B, 14-8D, 5-4B and 2-7B) were then detected using SK-Hep1 cells transfected with or without shNPC2. Among these NPC2 mAbs, we selected one NPC2 mAb (3-6B) as the best for the further research and use in clinical applications.

### Differential expression of NPC2 in human normal and tumor tissues

**Figure 3:**
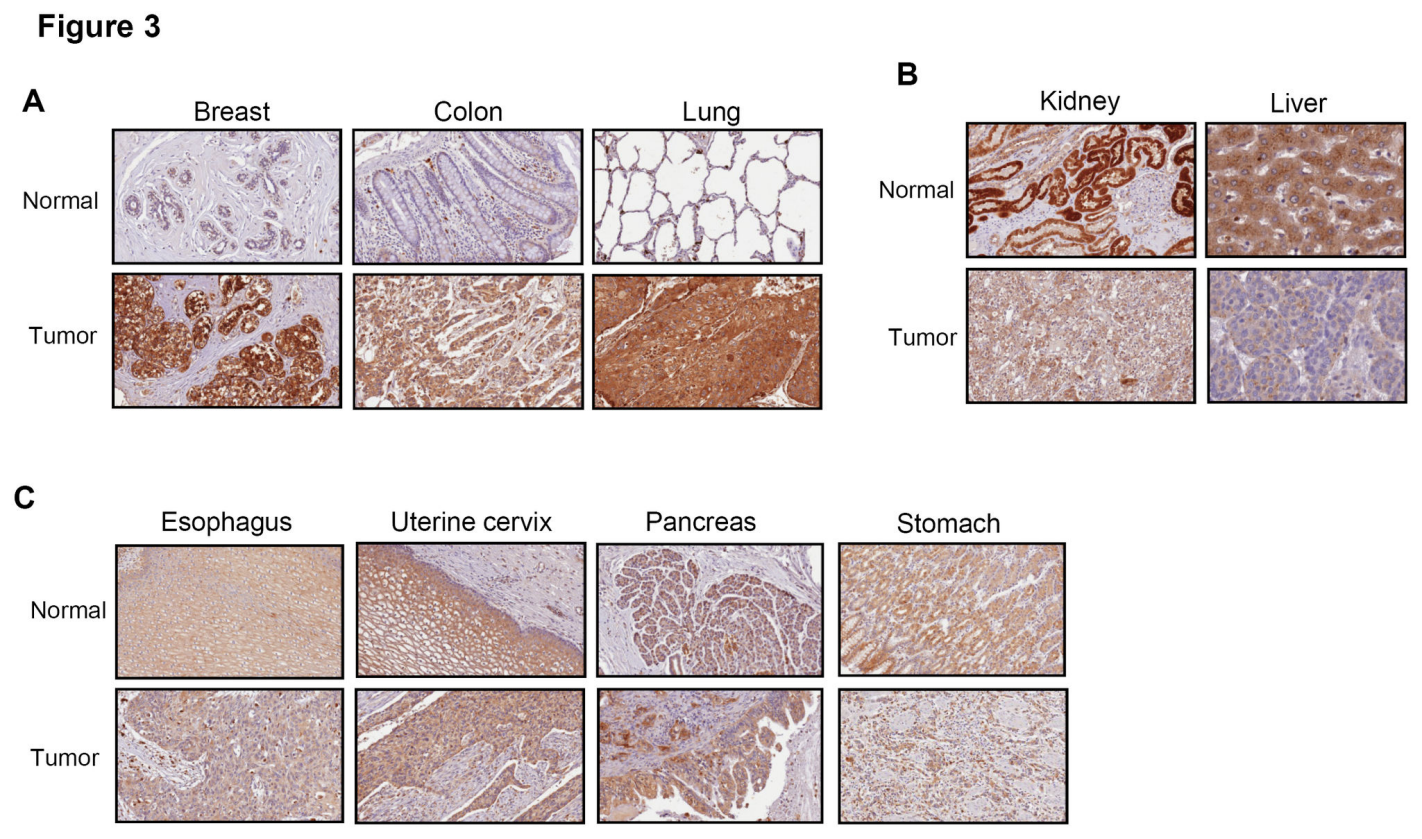
IHC staining of NPC2 protein expression in paired human tumor tissue samples and their normal counterparts. Magnification: 200x. (A) NPC2 was up-regulated in breast, colon and lung tumor samples. (B) NPC2 was down-regulated in kidney and liver tumor samples. (C) The expression of NPC2 remained unchanged in the pancreatic, esophageal, uterine cervical and stomach tissue sample pairs.

In order to investigate the clinical application of NPC2 mAb, we detected the expression of NPC2 in various normal and cancer tissues using IHC staining. Normal esophageal, uterine cervical, pancreatic, stomach, breast, colon and lung tissue showed moderate to weak intensity NPC2 staining (Figures 3A and 3C). As shown in Figure 3B, strong expression of NPC2 was found in normal hepatocytes of liver. In kidney, NPC2 was expressed both in the distal and proximal convoluted tubule, but not in the glomerulus (Figure 3B).

Comparison between these normal tissues (N) and their corresponding tumor tissues (T) was carried out and it was found that NPC2 was significantly up-regulated in breast, colon and lung cancers (Figure 3A). Among the 23 pairs of breast tumor and normal tissues tested, 18 (78%) of the tumor tissues showed higher NPC2 expression levels than their normal tissues (*p*<0.0001, Table 1). Out of 44 matched non-small cell lung carcinoma pairs, 30 tumor tissue samples (68%) had greater NPC2 expression than their normal counterparts (*p*=0.02, Table 1). In contrast, NPC2 was significantly down-regulated in renal cell carcinoma and liver cancer (Figure 3B). Among 33 pairs of kidney tumor and normal tissues, 31 (94%) tumor tissue samples had a much lower NPC2 expression than the equivalent normal tissue sample (*p*<0.0001, Table 1). Among 50 matched liver tumor tissue and tumor-adjacent tissue pairs, 36 tumor tissue samples (72%) showed much lower NPC2 expression than their tumor-adjacent tissue sample (*p*=0.02, Table 1). However, there were no difference between normal and cancer tissue samples for carcinoma of the esophagus, uterine cervix, pancreas, and stomach (Figure 3C). Taken together, these results indicated that aberrant expression of NPC2 is associated with different cancers and that the change in expression may be either up or down depending on the tissue.

**Table 1:** Immunohistochemistry results of NPC2 expression in human breast, colon, lung, kidney and liver cancer paired tissues.

**Cancers**	**T>N n (%**)	**T=N n (%)**	**T<N n (%)**	***p* value**
**Increase in tumor tissues**				
Breast (n=23)	18 (78%)	4 (17%)	1 (4%)	<0.0001
Colon (n=38)	18 (47%)	14 (37%)	6 (16%)	0.0523
Lung^#^ (n=44)	30 (68%)	5 (11%)	9 (20%)	0.02
**Decrease in tumor tissues**				
Kidney (n=33)	2 (6%)	0	31 (94%)	<0.0001
Liver (n=50)	2 (4%)	12 (24%)	36 (72%)	0.02

T, tumor tissues; N, normal tissues; ^#^, Non-small cell lung carcinoma.

### Relationship between NPC2 expression and the status of ER, PR and HER-2

Regarding to breast cancer, we further collected 33 invasive breast cancer subjects identified in the Kaohsiung Municipal Ta-Tung Hospital and analyzed the relationship between NPC2 expression and clinicopathological characteristics. Among 33 patients, 18 (54.5%) tumor tissue samples had a much higher NPC2 expression than the equivalent normal tissue sample (*p*=0.002, Table 2). Subjects with NPC2 up-regulation were more likely to have a later pathology stage (*p*=0.018), ER(-) (*p*=0.007), PR(-) (*p*=0.005), HER-2(+) (*p*=0.006), ER(-) plus PR(-) (*p*=0.011) and ER/PR(-) plus HER-2(+) (*p*=0.011).

**Table 2:** NPC2 expression and clinicopathological characteristics of breast cancer patients.

	**Total**	**T>N n (%)**	**T=N n (%)**	**T<N n (%)**	***p* value**
Total patients	33	18 (55%)	13 (39%)	2 (6%)	0.002
TNM stage					
I	9	5 (56%)	4 (44%)	0	0.043
II	16	6 (38%)	8 (50%)	2 (12%)	0.233
III	8	7 (88%)	1 (12%)	0	0.018
ER					
Positive	20	9 (45%)	9 (45%)	2 (10%)	0.090
Negative	13	9 (69%)	4 (31%)	0	0.007
PR					
Positive	18	8 (44%)	8 (44%)	2 (11%)	0.092
Negative	15	10 (67%)	5 (33%)	0	0.005
HER-2 Score					
Positive (3)	20	12 (60%)	7 (35%)	1 (5%)	0.006
Negative (0-2)	13	6 (46%)	6 (46%)	1 (8%)	0.091
Combination					
ER(+), PR(+)	16	7 (44%)	7 (44%)	2 (12%)	0.171
ER(+), PR(-)	4	2 (50%)	2 (50%)	0	0.180
ER(-), PR(+)	2	1 (50%)	1 (50%)	0	0.317
ER(-), PR(-)	11	8 (73%)	3 (27%)	0	0.011
ER/PR(+), HER-2(+)	9	4 (44%)	4 (44%)	1 (11%)	0.076
ER/PR(+), HER-2(-)	13	6 (46%)	6 (46%)	1 (8%)	0.091
ER/PR(-), HER-2(+)	11	8 (73%)	3 (27%)	0	0.011
ER/PR(-), HER-2(-)	0	0	0	0	N/A

T, tumor tissues; N, normal tissues; ER, estrogen receptor; PR, progesterone receptor; HER-2, human epidermal growth factor receptor 2; (+), positive; (-), negative.

### Dysregulation of NPC2 expression in human cirrhosis and HCC

**Figure 4:**
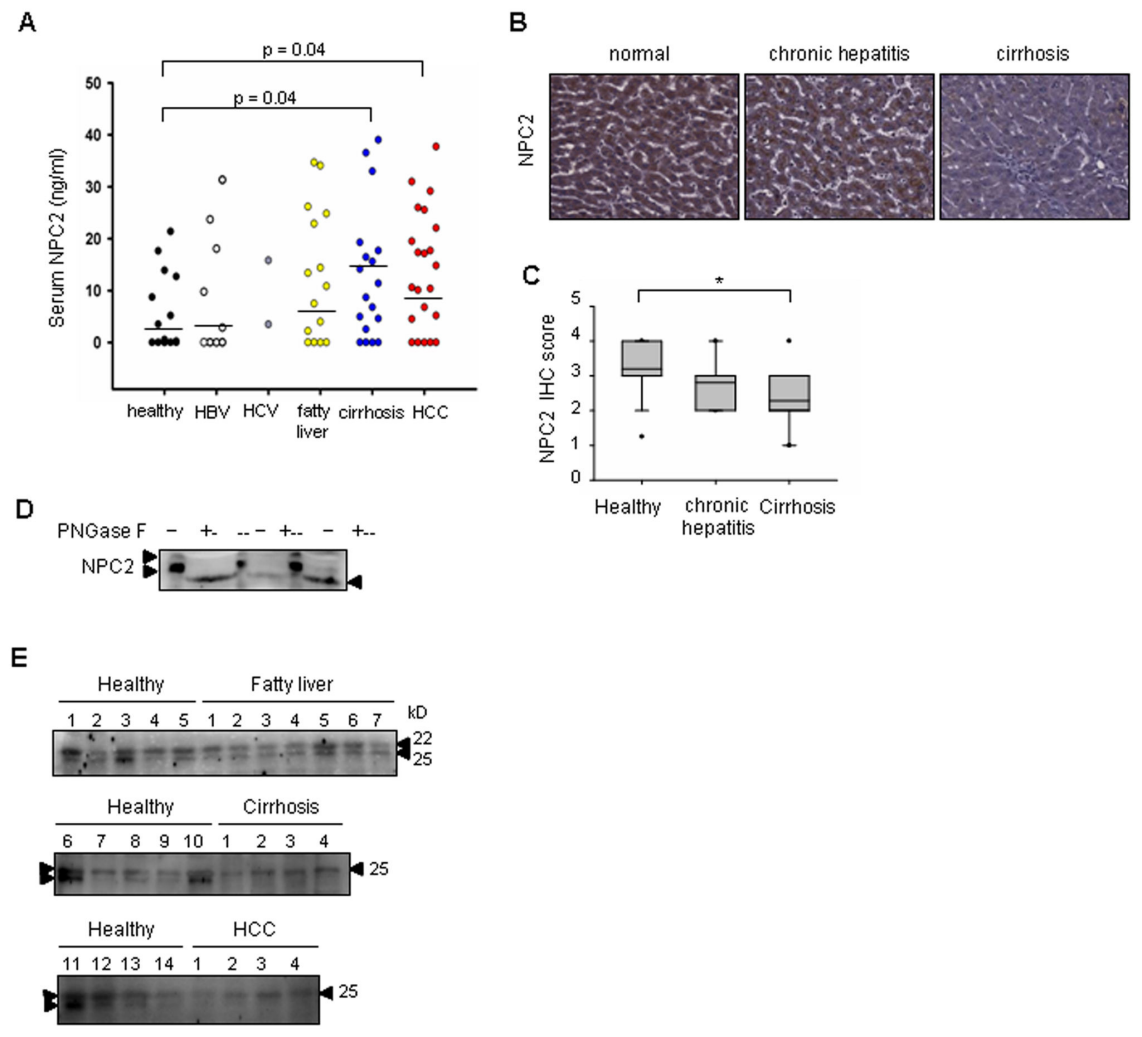
Dysregulation of NPC2 is associated with cirrhosis and HCC. (A) ELISA analysis of serum NPC2 levels in 42 healthy controls, 20 patients with chronic HBV infection, 2 patients with chronic HCV infection, 27 patients with fatty liver, 28 cirrhosis and 46 HCC patients. (B) IHC staining of NPC2 protein expression in normal, chronic hepatitis and cirrhosis tissues. (C) IHC analysis of hepatic NPC2 expression in 44 normal, 21 chronic hepatitis and 60 cirrhosis tissue samples (*, *p*<0.05, Mann-Whitney U test). (D) Human sera (60 μg) were incubated with peptide N-glycosidase F (PNGase F) and subjected to Western blot analysis. (E) Western blot analysis of serum NPC2 expression in 14 healthy controls, 7 fatty liver, 4 cirrhosis and 4 HCC patients. Each line was loaded 100 µg protein.

Since liver is the main source of plasma and biliary NPC2 [13], we next examined whether serum NPC2 level can be used as a novel biomarker for liver diseases. We measured the amount of serum NPC2 level from 42 healthy controls, 20 patients with HBV chronic infection, 2 patients with HCV chronic infection, 27 patients with fatty liver, 28 patients with cirrhosis and 46 HCC using an ELISA (Figure 4A). The levels of NPC2 in groups with healthy control, chronic HBV infection, chronic HCV infection, fatty liver, cirrhosis and HCC were 3.29 ± 1.43 ng/ml, 4.51 ± 1.88 ng/ml, 9.66 ng/ml (maximum value, 15.81 and minimum value, 3.504), 7.49 ± 2.30 ng/ml, 15.30 ± 2.02 ng/ml and 7.26 ± 2.11 ng/ml, respectively. As shown in Figure 4A, the levels of NPC2 were no difference between healthy control, chronic hepatitis (HBV and HCV) infection and patients with fatty liver disease. Importantly, patients with cirrhosis and HCC had significantly higher levels of NPC2 than healthy controls. On the other hand, the IHC staining demonstrated that NPC2 was significantly down-regulated in cirrhosis tissues, while there was no difference between normal and hepatitis tissues (Figures 4B and 4C).

Since N-glycosylation is important for the proper targeting and function of NPC2 [14], we next used PNGase F digestion to confirm whether serum NPC2 protein heterogeneity is due to post-translational glycosylation modification. After PNGase F treatment, NPC2 was visualized as a single immunoreactive band (Figure 4D). To further examined whether glycosylated form of NPC2 is associated with liver cancer progression, serum samples were subjected to western blotting analysis. Glycosylated and nascent NPC2 were expressed at a ratio of 1:1 in normal human sera (Figure 4E). Interestingly, although we did not find any difference in serum NPC2 expression between healthy and fatty liver patients, glycosylated NPC2 expression was the major form in both cirrhosis and HCC patients (Figure 4E). These data suggested that the amounts and the changes of glycosylated pattern of NPC2 are associated with cirrhosis and HCC development.

### Effects of NPC2 on MAPK signaling

**Figure 5:**
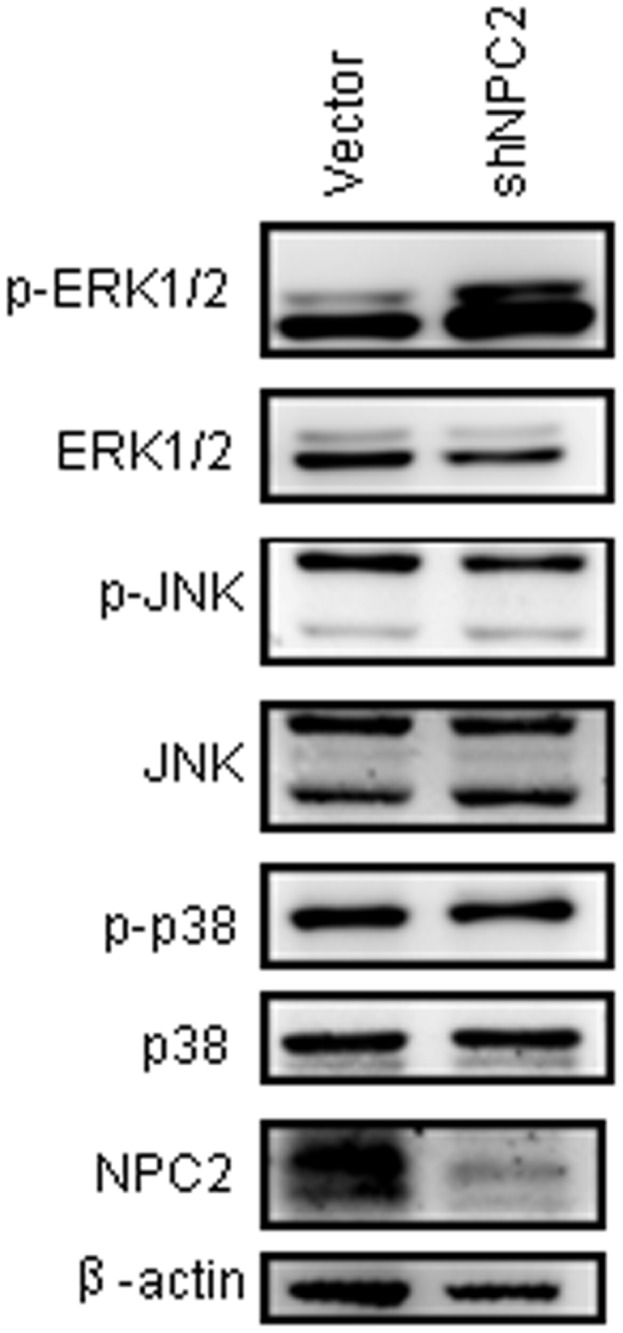
The effects of NPC2 on MAPK signaling. Total and phosphorylated ERK1/2, p38, JNK were detected by Western blotting from the SK-hep1 cells. Knockdown of NPC2 results in activation of ERK1/2.

To identify the NPC2-dependent mechanism, we emphasized on the status of MAPK signaling. As shown in Figure 5, sustained phosphorylation of ERK1/2 has been observed in NPC2 knockdown cells. However, p38 and JNK did not activate in NPC2 knockdown cells.

## Discussion

NPC2 protein binds with free cholesterol and controls the intracellular cholesterol homeostasis [2]. Mutation of the NPC2 gene results in cholesterol accumulation in the late endosomal/lysosomal compartment of cell [6]. However, the roles of NPC2 expression in human cancers are not fully understood notwithstanding its known role in cholesterol binding. In this study, we generated and characterized a number of NPC2 mAbs and then assessed their immunoreactivity using multiple tumor tissue arrays. From the epitope mapping data, we found that the NPC2 mAbs targeted amino acid residues 31-40 of the human NPC2 protein (Figure 2B). Since amino acid residues 31-40 are highly conserved among various mammalian orthologs [2], these NPC2 mAbs should be helpfully when carrying out research on the role of NPC2 in a wide range of mammalian species in the future. The sensitivity and specificity of NPC2 mAbs was evaluated by knock-down of NPC2 protein in SK-Hep1 cells (Figure 2C).

The IHC staining showed that NPC2 expression was increased in breast, colon and lung cancers (Figure 3A). Since strong expression of NPC2 is observed in human intraductal breast papilloma, colon polyps, lung papillary carcinoma and ovarian fimbria [8,15], NPC2 protein may contribute to papillae formation. Epidemiological studies demonstrated that HER-2 subtype [HER-2(+), ER(-), and PR(-)] and triple negative [HER-2(-), ER(-), and PR(-)] had the worst survival, poor prognosis and higher locoregional recurrence [16-19]. Our study found that NPC2 over-expression is associated with HER-2 subtype (Table 2), which suggests that NPC2 up-regulation may be a favorable prognosis predictor for breast cancer.

Cholesterol homeostasis is important to lamellar bodies of lung alveolar type II cells that serve to produce surfactant. NPC2 protein is present in the lung alveolar type II cells and alveolar macrophages and regulates surfactant cholesterol content [9,20]. Our findings showed that NPC2 is enhanced in lung adenocarcinoma tissue (Figure 3A). Proteomics analysis has also demonstrated that expression of NPC2 is increased in lung adenoma and pleural effusion [21,22]. Although it is not clear yet if NPC2 plays a role in lung adenocarcinoma, the presence of NPC2 protein in pleural effusion of patients with lung adenocarcinoma suggests that it may have a use as a potential diagnostic marker for lung cancer.

Reduced expression of NPC2 was specifically observed in kidney and liver tumor tissues, compared to their normal counterparts. Since NPC2 knockdown fibroblast cells have been shown a sustained phosphorylation of ERK1/2 MAPK [10], NPC2 may negatively regulate ERK1/2 MAPK activation. Indeed, the activation of ERK1/2 was observed in the NPC2 knockdown SK-hep1 cells (Figure 5). Regarding the effect of NPC2 replacement therapy in NPC2-/- mice, it was found that hepatic macrophage infiltration and the number of fat-laden cells were significantly improved [23]. Since lipid accumulation and subsequently inflammation accelerate the progression of cirrhosis and liver cancer [24,25], NPC2 administration may ameliorate the liver cancer development. Since liver is the main source of plasma and biliary NPC2 [13], the increase of sera NPC2 in cirrhosis and HCC patients may related to the severe damage of hepatocytes. In the present study we also observed changes in glycosylated NPC2 expression patterns in the sera of both cirrhosis and HCC patients, suggesting that such changes may serve as an indicator of liver cirrhosis and cancer. N-glycosylation is important for the proper targeting and function of NPC2 [14]. Further study is needed to clarify the roles of glycosylated NPC2 using more cirrhosis and HCC cases.

In the kidneys, NPC2 is abundantly express in distal and proximal convoluted tubule (Figure 3B). Since NPC2 is up-regulated in kidney tissues from Dahl salt-sensitive rat given a high-salt diet [26], it has been suggested that NPC2 might regulate sodium reabsorption in the nephron. Nevertheless, the functional relevance of NPC2 in renal cell carcinoma remains unclear and further studies are needed to elucidate the role of NPC2 in kidney, both physiologically and pathologically.

Our findings indicated that our NPC2 monoclonal antibody has potentially useful applications in the areas of clinical diagnosis and cancer progression across multiple tissues.

## Supporting Information

Table S1 **#, HBV or HCV indicate cases were infected by HBV and HCV virus, respectively.**
Non-B & non-C indicate patients were not infected by HBV and HCV virus. *, The classification of pathology stages were according to AJCC, UICC, and CUPI.
(DOC)

Table S2 **#, HBV or HCV indicate cases were infected by HBV and HCV virus, respectively.**
(DOC)

## References

[1] Kirchhoff C, Osterhoff C, Young L (1996) Molecular cloning and characterization of HE1, a major secretory protein of the human epididymis. Biol Reprod 54: 847-856. doi:10.1095/biolreprod54.4.847. PubMed: 8924505. 8924505

[2] Storch J, Xu Z (2009) Niemann-Pick C2 (NPC2) and intracellular cholesterol trafficking. Biochim Biophys Acta 1791: 671-678. doi:10.1016/j.bbalip.2009.02.001. PubMed: 19232397. 19232397 PMC4281484

[3] Mukherjee S, Maxfield FR (2004) Lipid and cholesterol trafficking in NPC. Biochim Biophys Acta 1685: 28-37. doi:10.1016/j.bbalip.2004.08.009. PubMed: 15465424. 15465424

[4] Naureckiene S, Sleat DE, Lackland H, Fensom A, Vanier MT et al. (2000) Identification of HE1 as the second gene of Niemann-Pick C disease. Science 290: 2298-2301. doi:10.1126/science.290.5500.2298. PubMed: 11125141. 11125141

[5] Sleat DE, Wiseman JA, El-Banna M, Price SM, Verot L et al. (2004) Genetic evidence for nonredundant functional cooperativity between NPC1 and NPC2 in lipid transport. Proc Natl Acad Sci U S A 101: 5886-5891. doi:10.1073/pnas.0308456101. PubMed: 15071184. 15071184 PMC395893

[6] Vanier MT, Millat G (2004) Structure and function of the NPC2 protein. Biochim Biophys Acta 1685: 14-21. doi:10.1016/j.bbalip.2004.08.007. PubMed: 15465422. 15465422

[7] Liao YJ, Chen TL, Lee TS, Wang HA, Wang CK et al. (2012) Glycine N-methyltransferase deficiency affects niemann-pick type c2 protein stability and regulates hepatic cholesterol homeostasis. Mol Med 18: 412-422. PubMed: 22183894. 22183894 10.2119/molmed.2011.00258 PMC3356423

[8] Sugawara M, Ohye H, Tomoda C, Kogai T, Kamata Y et al. (2011) A novel role for Niemann-Pick disease type 2C protein in papillae formation. PLOS ONE 6: e15777. doi:10.1371/journal.pone.0015777. PubMed: 21253586. 21253586 PMC3017059

[9] Roszell BR, Tao JQ, Yu KJ, Huang S, Bates SR (2012) Characterization of the Niemann-Pick C pathway in alveolar type II cells and lamellar bodies of the lung. Am J Physiol Lung Cell Mol Physiol 302: L919-L932. doi:10.1152/ajplung.00383.2011. PubMed: 22367786. 22367786 PMC3362154

[10] Csepeggi C, Jiang M, Kojima F, Crofford LJ, Frolov A (2011) Somatic cell plasticity and Niemann-Pick type C2 protein: fibroblast activation. J Biol Chem 286: 2078-2087. doi:10.1074/jbc.M110.135897. PubMed: 21084287. 21084287 PMC3023505

[11] Liu HH, Chen KH, Shih YP, Lui WY, Wong FH et al. (2003) Characterization of reduced expression of glycine N-methyltransferase in cancerous hepatic tissues using two newly developed monoclonal antibodies. J Biomed Sci 10: 87-97. doi:10.1007/BF02256001. PubMed: 12566990. 12566990

[12] Chu TM, Kawinski E, Lin TH (1993) Characterization of a new monoclonal antibody F4 detecting cell surface epitope and P-glycoprotein in drug-resistant human tumor cell lines. Hybridoma 12: 417-429. doi:10.1089/hyb.1993.12.417. PubMed: 7503940. 7503940

[13] Klein A, Amigo L, Retamal MJ, Morales MG, Miquel JF et al. (2006) NPC2 is expressed in human and murine liver and secreted into bile: potential implications for body cholesterol homeostasis. Hepatology 43: 126-133. doi:10.1002/hep.20985. PubMed: 16374838. 16374838

[14] Chikh K, Vey S, Simonot C, Vanier MT, Millat G (2004) Niemann-Pick type C disease: importance of N-glycosylation sites for function and cellular location of the NPC2 protein. Mol Genet Metab 83: 220-230. doi:10.1016/j.ymgme.2004.06.013. PubMed: 15542393. 15542393

[15] Asakawa J, Kodaira M, Ishikawa N, Hirai Y, Nagataki S et al. (2002) Two-dimensional complementary deoxyribonucleic acid electrophoresis revealing up-regulated human epididymal protein-1 and down-regulated CL-100 in thyroid papillary carcinoma. Endocrinology 143: 4422-4428. doi:10.1210/en.2002-220550. PubMed: 12399439. 12399439

[16] Lowery AJ, Kell MR, Glynn RW, Kerin MJ, Sweeney KJ (2012) Locoregional recurrence after breast cancer surgery: a systematic review by receptor phenotype. Breast Cancer Res Treat 133: 831-841. doi:10.1007/s10549-011-1891-6. PubMed: 22147079. 22147079

[17] Caldarella A, Crocetti E, Bianchi S, Vezzosi V, Urso C et al. (2011) Female breast cancer status according to ER, PR and HER2 expression: a population based analysis. Pathol Oncol Res 17: 753-758. doi:10.1007/s12253-011-9381-z. PubMed: 21479875. 21479875

[18] Onitilo AA, Engel JM, Greenlee RT, Mukesh BN (2009) Breast cancer subtypes based on ER/PR and Her2 expression: comparison of clinicopathologic features and survival. Clin Med Res 7: 4-13. doi:10.3121/cmr.2008.825. PubMed: 19574486. 19574486 PMC2705275

[19] Carey LA, Perou CM, Livasy CA, Dressler LG, Cowan D et al. (2006) Race, breast cancer subtypes, and survival in the Carolina Breast Cancer Study. JAMA 295: 2492-2502. doi:10.1001/jama.295.21.2492. PubMed: 16757721. 16757721

[20] Griese M, Brasch F, Aldana VR, Cabrera MM, Goelnitz U et al. (2010) Respiratory disease in Niemann-Pick type C2 is caused by pulmonary alveolar proteinosis. Clin Genet 77: 119-130. doi:10.1111/j.1399-0004.2009.01325.x. PubMed: 20002450. 20002450

[21] Pernemalm M, De Petris L, Eriksson H, Brandén E, Koyi H et al. (2009) Use of narrow-range peptide IEF to improve detection of lung adenocarcinoma markers in plasma and pleural effusion. Proteomics 9: 3414-3424. doi:10.1002/pmic.200800814. PubMed: 19609957. 19609957

[22] Taguchi A, Politi K, Pitteri SJ, Lockwood WW, Faça VM et al. (2011) Lung cancer signatures in plasma based on proteome profiling of mouse tumor models. Cancer Cell 20: 289-299. doi:10.1016/j.ccr.2011.08.007. PubMed: 21907921. 21907921 PMC3406925

[23] Nielsen GK, Dagnaes-Hansen F, Holm IE, Meaney S, Symula D et al. (2011) Protein replacement therapy partially corrects the cholesterol-storage phenotype in a mouse model of Niemann-Pick type C2 disease. PLOS ONE 6: e27287. doi:10.1371/journal.pone.0027287. PubMed: 22073306. 22073306 PMC3207855

[24] Starley BQ, Calcagno CJ, Harrison SA (2010) Nonalcoholic fatty liver disease and hepatocellular carcinoma: a weighty connection. Hepatology 51: 1820-1832. doi:10.1002/hep.23594. PubMed: 20432259. 20432259

[25] Day CP (2002) Pathogenesis of steatohepatitis. Best Pract Res Clin Gastroenterol 16: 663-678. doi:10.1053/bega.2002.0333. PubMed: 12406438. 12406438

[26] Araki N, Ishigami T, Ushio H, Minegishi S, Umemura M et al. (2009) Identification of NPC2 protein as interaction molecule with C2 domain of human Nedd4L. Biochem Biophys Res Commun 388: 290-296. doi:10.1016/j.bbrc.2009.07.158. PubMed: 19664597. 19664597

