# Supplementary material for: Characterization of Niemann-Pick Type C2 Protein Expression in Multiple Cancers Using a Novel NPC2 Monoclonal Antibody
Source: PLoS One. 2013 Oct 17;8(10):e77586. doi: 10.1371/journal.pone.0077586 (PMC3798307; doi:10.1371/journal.pone.0077586)
Supplement: Table S1 — #, HBV or HCV indicate cases were infected by HBV and HCV virus, respectively. Non-B & non-C indicate patients were not infected by HBV and HCV virus. *, The classification of pathology stages were according to AJCC, UICC, and CUPI. (DOC) [file pone.0077586.s001.doc]

Table S1. Detailed information of 50 HCC cases

| Case number | gender | age | tumor size (cm) | viral status^#^ | Pathology stage* | cirrhosis |
| --- | --- | --- | --- | --- | --- | --- |
| 1 | male | 31 | 17 | HBV | IIIA | yes |
| 2 | male | 68 | 3.5 | HBV | I | no |
| 3 | male | 33 | 2.5 | HBV | II | no |
| 4 | male | 54 | 2.5 | HBV | I | yes |
| 5 | male | 53 | 11 | HBV | IIIA | no |
| 6 | male | 64 | 6.5 | HBV | I | yes |
| 7 | male | 47 | 2.5 | HBV | II | yes |
| 8 | male | 37 | 3 | HBV | II | yes |
| 9 | female | 62 | 4 | HBV | I | yes |
| 10 | female | 56 | 4.8 | HBV | I | no |
| 11 | female | 43 | 5 | HBV | II | no |
| 12 | female | 28 | 9.5 | HBV | II | yes |
| 13 | female | 30 | 13 | HBV | IV | no |
| 14 | female | 71 | 6 | HBV | IIIA | yes |
| 15 | female | 68 | 3.2 | HBV | IV | yes |
| 16 | female | 72 | 5 | HBV | II | yes |
| 17 | female | 69 | 5 | HBV | IIIA | yes |
| 18 | female | 67 | 4 | HBV | I | no |
| 19 | male | 74 | 6 | HCV | II | no |
| 20 | male | 72 | 3 | HCV | I | yes |
| 21 | male | 63 | 6 | HCV | I | yes |
| 22 | male | 68 | 2.5 | HCV | I | yes |
| 23 | male | 52 | 2.5 | HCV | II | yes |
| 24 | male | 70 | 3.7 | HCV | I | yes |
| 25 | male | 63 | 2.2 | HCV | I | no |
| 26 | male | 65 | 3.5 | HCV | II | yes |
| 27 | female | 48 | 3.5 | HCV | I | no |
| 28 | female | 77 | 5.5 | HCV | II | yes |
| 29 | female | 59 | 3 | HCV | I | yes |
| 30 | female | 57 | 4 | HCV | I | yes |
| 31 | female | 65 | 3.5 | HCV | I | yes |
| 32 | female | 85 | 12 | HCV | I | no |
| 33 | female | 62 | 2 | HCV | I | yes |
| 34 | female | 55 | 4.3 | HCV | II | yes |
| 35 | male | 61 | 5 | non-B & non-C | II | yes |
| 36 | male | 55 | 4.5 | non-B & non-C | I | no |
| 37 | male | 74 | 4.5 | non-B & non-C | II | yes |
| 38 | male | 63 | 3 | non-B & non-C | I | yes |
| 39 | male | 61 | 4.5 | non-B & non-C | I | yes |
| 40 | male | 71 | 5 | non-B & non-C | II | no |
| 41 | male | 58 | 9 | non-B & non-C | IIIA | no |
| 42 | male | 63 | 5.5 | non-B & non-C | I | yes |
| 43 | male | 73 | 4.3 | non-B & non-C | I | no |
| 44 | male | 67 | 5.2 | non-B & non-C | I | no |
| 45 | female | 72 | 8 | non-B & non-C | I | no |
| 46 | female | 75 | 10.5 | non-B & non-C | IIIA | no |
| 47 | female | 66 | 6.6 | non-B & non-C | IV | no |
| 48 | female | 51 | 11.5 | non-B & non-C | IIIA | no |
| 49 | female | 31 | 24.3 | non-B & non-C | IIIA | no |
| 50 | female | 50 | 11 | non-B & non-C | II | yes |

#, HBV or HCV indicate cases were infected by HBV and HCV virus, respectively. Non-B & non-C indicate patients were not infected by HBV and HCV virus.

*, The classification of pathology stages were according to AJCC, UICC, and CUPI.
