# Supplementary material for: Characterization of Niemann-Pick Type C2 Protein Expression in Multiple Cancers Using a Novel NPC2 Monoclonal Antibody
Source: PLoS One. 2013 Oct 17;8(10):e77586. doi: 10.1371/journal.pone.0077586 (PMC3798307; doi:10.1371/journal.pone.0077586)
Supplement: Table S2 — #, HBV or HCV indicate cases were infected by HBV and HCV virus, respectively. (DOC) [file pone.0077586.s002.doc]

Table S2. Detailed information of 165 patients with different liver diseases

|  | Healthy | HBV carrier | HCV carrier | Fatty liver | Cirrhosis | HCC |
| --- | --- | --- | --- | --- | --- | --- |
| N (%) | 42 (25.5%) | 20 (12.1%) | 2 (1.2%) | 27 (16.4) | 28 (16.9) | 46 (27.8%) |
| Age | 70.4±18.9 | 47.5±14.1 | 51 | 53.3±18.6 | 59.3±12.4 | 65.4±12.7 |
| Gender |  |  |  |  |  |  |
| male | 26 (61.9%) | 12 (60.0%) | 1 (50.0%) | 20 (74.1%) | 19 (67.8%) | 35 (76.1%) |
| female | 16 (38.1%) | 8 (40.0%) | 1 (50.0%) | 7 (25.9%) | 9 (32.2%) | 11 (23.9%) |
